# A Throughfall Collection Method Using Mixed Bed Ion Exchange Resin Columns

**DOI:** 10.1100/tsw.2002.84

**Published:** 2002-01-15

**Authors:** Mark E. Fenn, Mark A. Poth, Michael J. Arbaugh

**Affiliations:** USDA Forest Service, Pacific Southwest Research Station, Forest Fire Laboratory, 4955 Canyon Crest Drive, Riverside, CA 92507-6099, USA; USDA-CSREES, National Research Initiative Competitive Grants Program, Stop 2241, 1400 Independence Ave. SW, Washington, D.C. 20250-2241, USA

**Keywords:** ion exchange resin, nitrogen deposition, throughfall, throughfall collector

## Abstract

Measurement of ionic deposition in throughfall is a widely used method for measuring deposition inputs to the forest floor. Many studies have been published, providing a large database of throughfall deposition inputs to forests. However, throughfall collection and analysis is labor intensive and expensive because of the large number of replicate collectors needed and because sample collection and chemical analyses are required on a stochastic precipitation event-based schedule. Therefore we developed and tested a throughfall collector system using a mixed bed ion exchange resin column. We anticipate that this method will typically require only one to three samplings per year. With this method, bulk deposition and bulk throughfall are collected by a funnel or snow tube and ions are retained as the solution percolates through the resin column. Ions retained by the resin are then extracted in the same column with 2N KCl and analyzed for nitrate and ammonium. Deposition values in throughfall from conventional throughfall solution collectors and colocated ion exchange samplers were not significantly different during consecutive 3- and 4-month exposure periods at a high (Camp Paivika; >35 kg N ha^-1^ year^-1^) and a low deposition (Barton Flats; 5–9 kg N ha^-1^ year^-1^) site in the San Bernardino Mountains in southern California. N deposition in throughfall under mature pine trees at Camp Paivika after 7 months of exposure was extremely high (87 and 92 kg ha^-1^ based on the two collector types) compared to Barton Flats (11 and 13 kg ha^-1^). A large proportion of the N deposited in throughfall at Camp Paivika occurred as fog drip, demonstrating the importance of fog deposition as an input source of N at this site. By comparison, bulk deposition rates in open areas were 5.1 and 5.4 kg ha^-1^ at Camp Paivika based on the two collector types, and 1.9 and 3.0 kg ha^-1^ at Barton Flats.

Measurement of nutrient deposition in throughfall is a widely used method for measuring deposition inputs to the forest floor. Many studies have been published, providing a large database of throughfall deposition inputs to forests[1,2]. However, throughfall collection and analysis is labor intensive and expensive because of the large number of replicate collectors needed and because sample collection and chemical analyses are required on a stochastic precipitation event-based schedule. The use of ion exchange resin for capture of ions deposited in precipitation and throughfall would greatly reduce the number of collections and sample analyses required to measure deposition inputs. In a few published studies, ion exchange resin bags have been used to measure throughfall deposition or snowmelt fluxes of selected ions[3,4,5,6,7,8]. In a recent study in Sweden, throughfall deposition determined with ion exchange resin bags was highly correlated with throughfall inputs measured with colocated conventional throughfall collectors, suggesting the viability of the ion exchange resin method of measuring throughfall[3]. Ion exchange throughfall collectors can be considered as passive throughfall samplers, analogous to the use of passive samplers for monitoring gaseous pollutants[9], except that the field exposure times for passive throughfall collectors are much greater and throughfall collection is more stochastic than gaseous pollutant exposures.

The primary objective of this study was to test a modification of the resin bag throughfall technique by funneling precipitation or throughfall solutions through columns containing ion exchange resin. At least one other research group has also been developing similar ion exchange resin columns to measure ionic deposition in throughfall (Kathy Weathers, personal communication). We recently became aware of previous studies using ion exchange resin columns to collect ammonium and nitrate in rain and throughfall[10], but the objective of these studies was to characterize the isotopic composition of the atmospheric N collected by the resin columns. Compared to the resin bag technique, we expected the resin column design to improve flow of the solutions through the resin bed and to increase the contact of the solutions with the resin, thus improving ion capture. The throughfall collector system reported herein employs a column filled with mixed bed (anion + cation) ion exchange resin. We anticipate that this method will typically require only one to three samplings per year. With this method, it is not necessary to collect the throughfall or bulk deposition solution, as the ions of interest are captured on the ion exchange resin. After sample collection in the field, ions retained by the resin can be extracted in the same column with 2 *N* KCl and analyzed for nitrate and ammonium[3]. In this report, results from consecutive 3- and 4-month exposures at a high and a low deposition site in the San Bernardino Mountains (SBM) will be compared to results from conventional throughfall collectors colocated with the ion exchange samplers.

## EXPERIMENTAL METHODS/PROCEDURES

### Laboratory Tests and Methodology

The resin used for the ion exchange collectors is a mixed bed anion and cation exchange resin (Amberlite™ MB150);[a] 35 g of the resin were added to each column (1.27-cm diameter PVC tubing) and rinsed with distilled water. This is the same amount of resin used in a previous study[3]. Based on previous throughfall studies in the SBM[11] and the ion exchange capacity of the resin, we determined that 35 g of ion exchange resin is in excess of the ion exchange capacity needed for throughfall collection at the study sites. Polyester floss was inserted at the bottom and top of the resin columns and the bottom closed with a slit cap to allow the throughfall solution to percolate through the column. Throughfall solution or precipitation samples were collected with

[a] Mention of trade names or products is for information only and does not imply endorsement by the U.S. Department of Agriculture.

the same type of funnels used for the conventional throughfall samplers, except that the solution was allowed to drain through the resin column where the ions are adsorbed by the ion exchange resin.

Background levels of nitrate and ammonium in the resin columns were determined by extracting the columns three times with 200 ml of 2 *N* KCl. The total $NO_3$-N extracted was 0.01 mg/35 mg resin and 0.04 mg $NH_4$-N/35 mg resin. Considering the surface area of the throughfall collectors, this amount of background nitrate in the resin is equivalent to deposition of 0.01 kg N $ha^{-1,}$ and the background ammonium in the resin is equivalent to 0.05 kg N $ha^{-1}$.

In many studies, ion exchange resin is placed in nylon mesh bags for adsorption of ions from soil solution, snow melt, or atmospheric deposition[3,4,5,6,7,8]. Advantages of the ion exchange resin column design used in this study are that the resin is not in contact with the soil or forest floor, contact of the solution with the ion exchange resin is expected to be greater, and the resin can be easily extracted from the same columns used during the field exposure by percolating 200 ml of 2 *N* KCl solution through the intact resin columns. In laboratory trials we found that prerinsing the columns with 100 ml of distilled water prior to KCl extractions results in a more efficient and consistent extraction.

The columns were extracted three times (200 ml per extraction), although it soon became apparent that the amount of N removed in the third extraction was insignificant. Of the N in the three sequential extractions, only 0.07% of the nitrate and 0.1% of the ammonium was measured in the third extraction. The KCl extracts were analyzed for nitrate and ammonium on a TRAACS autoanalyzer. In nearly all of the first extracts, concentrations of nitrate and ammonium were many times greater than analysis detection limits. In the second and particularly in the third extracts, concentrations of ammonium and nitrate were often near detection limits.

Laboratory tests were performed with resin columns that were preloaded with a simulated throughfall solution, which was equivalent to a deposition rate of 70 kg $NO_3$-N $ha^{-1}$ and 36 kg $NH_4$-N $ha^{-1}$, in order to test the recovery efficiency of the adsorbed ions. Five columns were preloaded with the ionic solution and the experiment was repeated with similar results. This preloading rate is equal to more than double the N deposition rate over 3–4 months at Camp Paivika under the large pine trees used in this study. In these laboratory tests 97.9–103.9% of the nitrate and ammonium were extracted in the first extraction (Table 1). Multiple extractions of field-exposed but not preloaded columns, however, revealed that typically 91–94% of the nitrate is extracted in the first extraction, compared to 98–99% in the case of ammonium.

**TABLE 1**
**Laboratory Test of Ion Extraction Procedure from Ion Exchange Resin Columns. Resin Columns were Preloaded with a Known Amount of Nitrate and Ammonium in a Simulated Throughfall Solution***

| | ION RECOVERY FROM RESIN COLUMNS | | | | | |
|---|---|---|---|---|---|---|
| | After First Extraction | | After Second Extraction | | After Third Extraction | |
| | $NO_3$ | $NH_4$ | $NO_3$ | $NH_4$ | $NO_3$ | $NH_4$ |
| mg N: | 53.9 (1.1) | 29.3 (1.1) | 57.5 (1.2) | 29.5 (1.1) | 57.7 (1.2) | 29.5 (1.1) |
| Percent: | 97.9 | 103.9 | 104.4 | 104.5 | 104.9 | 104.5 |

*Average amount of $NO_3$-N added to each column = 55.1 mg. Average amount of $NH_4$-N added to each column = 28.2 mg. Each extraction consisted of 200 ml 2 *N* KCl percolated through the ion resin columns. Values in parentheses are standard errors of the mean (n = 5).

## Field Installation of the Throughfall Collectors

Throughfall collectors were installed at two sites in the SBM. Camp Paivika (CP; >35 kg N $ha^{-1}$ $year^{-1}$) is located on the western end of the SBM, and Barton Flats (BF; 5–9 kg N $ha^{-1}$ $year^{-1}$) is 45 km to the east of CP[11,12]. Colocated ion exchange resin collectors and conventional throughfall collectors (the liquid sample is collected) were placed under the north, east, south, and west quadrants of five large ponderosa or Jeffrey pine trees at each site. Because of the semiarid climate of these forests, the canopies do not have significant epiphytic communities. The colocated ion exchange resin and conventional collectors were placed 0.1–0.5 m apart in the field. In addition, colocated ion exchange and conventional collectors were placed in four canopy-free open areas at each site.

Throughfall samples from the conventional samplers were collected on a precipitation-event basis, while the ion exchange samplers were collected every 3 or 4 months. Collectors at BF were in place from July 6, 2000 to September 28, 2000 for the first exposure and from September 28, 2000 to February 8, 2001 for the second exposure. Analogous dates at CP were from July 7, 2000 to October 3, 2000, and October 3, 2000 to January 25, 2001. Comparisons of deposition values between colocated conventional and ion exchange collectors for each exposure period were tested for significant differences ($p \leq 0.05$) with paired t-tests (n = 5). Additional sets of ion exchange collectors will be left in the field for the next 3 months, for 6 months, for 9 months, and for a year in order to compare the effectiveness of the collectors with longer exposure times. One goal of this study was to determine how efficiently the resin collectors adsorb nitrate and ammonium under ambient montane conditions in California, and in particular, during the hot, dry summers characteristic of the area. Some studies suggest that air drying of resin may reduce their effectiveness in adsorbing inorganic N[13].

Both types of collectors were mounted on iron fence posts, with the collector opening ca. 1.5 m above ground level (Figs. 1 and 2). During snow-free periods all weather rain gauges (Productive Alternatives, Fergus Falls, MN) were used for the collection of throughfall for both types of collectors. The diameter of the funnel opening for these collectors is 10.0 cm[11,12]. An inert plastic screen (0.05-mm mesh size) was placed in the collector opening to exclude debris. Evidence of contamination from bird excrement, etc., was rare. During the summer, a white PVC cylinder (7.4-cm I.D.) was placed over the resin column to prevent direct solar exposure of the resin column. In winter, 7.4-cm diameter cylinders (1 m in length) were placed above the funnel collectors to allow for collection of snow. Fig. 1 shows the passive throughfall collectors with snow tubes attached to the top of the collector. Fig. 2 shows a close-up view of the bottom portion of the collector in winter (resin column is uncovered).

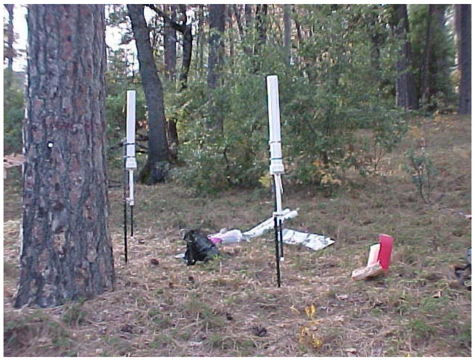

**FIGURE 1**. Ion exchange resin column throughfall collectors installed in the field. The smaller dark-colored tube at the bottom is the resin column. The large white cylinders at the top are the snow collector tubes. In summer, the snow collector tube is removed and a similar white cylinder is placed over the resin column to reduce direct exposure to solar radiation.

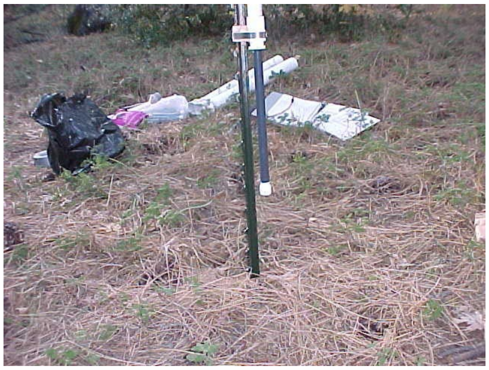

**FIGURE 2**. Close-up view of the ion exchange resin column in a typical winter installation.

## RESULTS AND DISCUSSION

### Precipitation and Throughfall Volumes

In each of the three sample collection dates at CP during the first 3 months (July–September) of the study, nearly all of the precipitation events were characterized by dense fog with little or no measurable incident precipitation in the open collectors. Only 17 mm of precipitation were collected in the open area collectors, while 154 mm of throughfall (predominantly fog drip) was collected under pine trees at CP. In contrast, at BF there were five precipitation events during the first 3 months, which were mainly characterized as low volume rainfall events (a total of 57 mm). On the first collection date at CP, only traces of precipitation were found in the open collectors and no sample was collected in the open area collectors on the second throughfall collection date. Such low sample volumes inherently lead to high variability in determining bulk deposition. With only trace amounts of precipitation, the conventional collectors typically do not collect enough sample for analysis, whereas the ion exchange collectors will retain ions from any solution transported to the resin.

Thus, differences in bulk deposition data between the two collector types does not necessarily mean that the ion exchange resin collectors did not perform adequately, but seems to be more a function of low sample volumes and inherent variability in bulk deposition measurements during fog episodes or low precipitation events. On the other hand, the tree canopies function as fog collectors resulting in adequate throughfall sample volumes. Consequently, both the conventional throughfall solution collectors and the ion exchange resin throughfall collectors gave similar deposition values during fog exposures and rain events.

During the second exposure period (October–January), precipitation events at CP were not as completely fog dominated as during the first 3 months. Precipitation volumes collected in the open area collectors during the entire 7 months of this study were 155 mm at BF and 249 mm at CP. Volumes collected under tree canopies were 129 and 784 mm at BF and CP, respectively. Thus, precipitation and throughfall volumes were 61 and 608% higher at CP than at BF over the 7 months. However, throughfall volume at CP was more than three times as great as precipitation in open areas at CP. This is due to the predominance of fog incidence at CP as noted above. The large canopies of the mature ponderosa pine trees chosen for this study served as effective scavengers of fog, leading to large volumes collected as fog drip in the throughfall collectors under these trees.

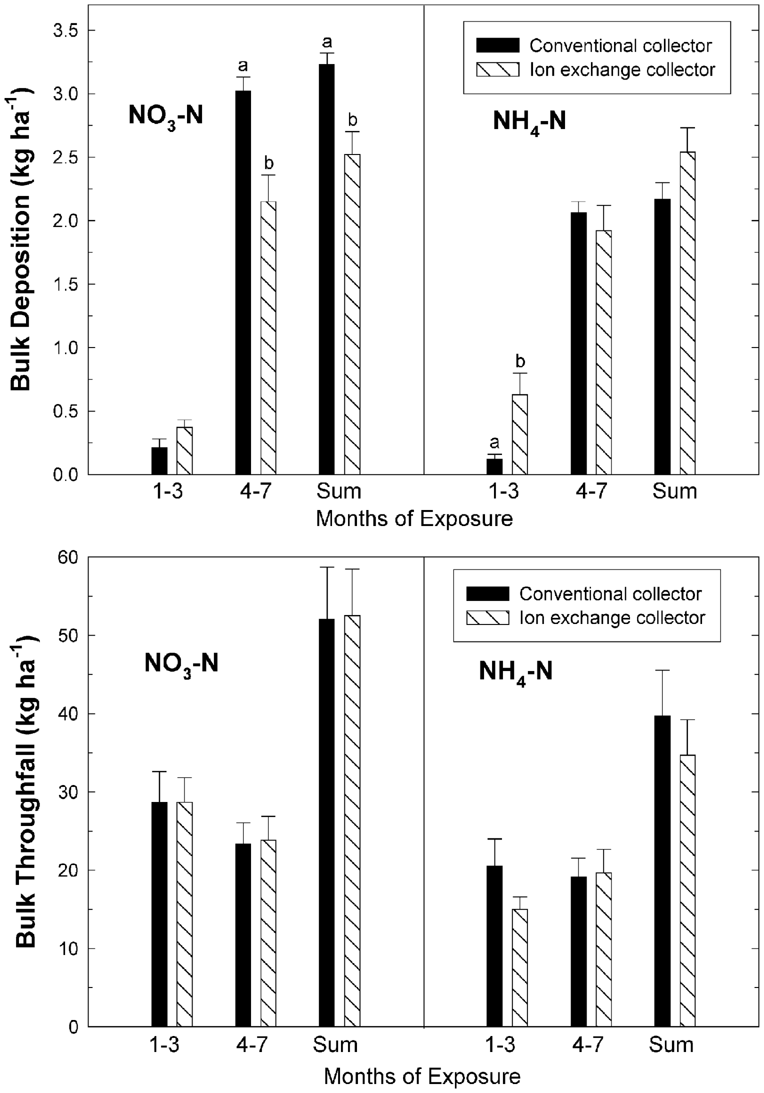

**FIGURE 3**. Comparison of deposition of $NO_3$-N and $NH_4$-N to conventional throughfall solution collectors and ion exchange resin collectors under pine trees and in open areas (bulk deposition) at Camp Paivika, a high deposition site in the San Bernardino Mountains. The first set of collectors was exposed for 3 months, followed by another set exposed for 4 months. Sum refers to total deposition during the two exposure periods combined. Letters above the error bars indicate significant differences ($p < 0.05$) in deposition between the collector types for that exposure period (n = 20 for throughfall collectors and n = 4 for bulk deposition collectors in open areas).

## Nitrogen Deposition Fluxes

At both field sites, average throughfall deposition fluxes of nitrate and ammonium under large pine canopies for two consecutive 3- or 4-month field exposure periods were not statistically different between the conventional throughfall and ion exchange throughfall collectors (Figs. 3 and 4). However, in 5 of 12 instances (combinations of ion and exposure period), significant differences ($p \leq 0.05$) were found in levels of bulk deposition (in open canopy-free areas) at the

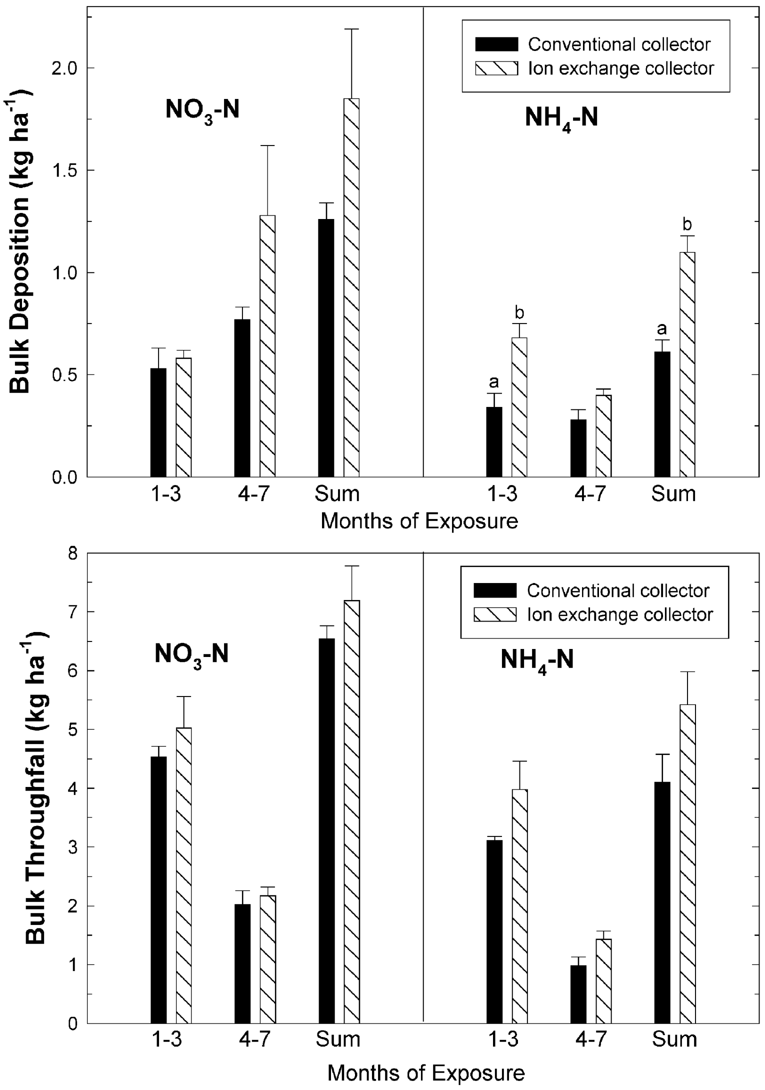

**FIGURE 4**. Comparison of deposition of $NO_3$-N and $NH_4$-N to conventional throughfall solution collectors and ion exchange resin collectors under pine trees and in open areas (bulk deposition) at Barton Flats, a low deposition site in the San Bernardino Mountains. The first set of collectors was exposed for 3 months, followed by another set exposed for 4 months. Sum refers to total deposition during the two exposure periods combined. Letters above the error bars indicate significant differences ($p < 0.05$) in deposition between the collector types for that exposure period (n = 20 for throughfall collectors and n = 4 for bulk deposition collectors in open areas).

two sites. In two instances, values were higher in the conventional collectors and in three instances deposition was higher in the ion exchange resin collectors. The factors thought to have resulted in differences in bulk deposition between the two methods include the following: lower replication of bulk deposition samplers (n = 4) compared to the throughfall collectors (n = 20), missing data due to animal disturbance of the open collectors on some dates, and low precipitation volumes. As a result of the low precipitation volumes at CP during the first 3-month exposure, bulk deposition was very low as determined by either method (Fig. 3).

As expected, deposition fluxes in throughfall were much greater at CP compared to BF (Figs. 3 and 4). Concentrations of nitrate in fog drip from the canopy at CP exceeded 1000 ppm in a few samples. The high N concentrations in fog in combination with high volumes of fog drip collected under the canopy resulted in very high fog deposition of N for the three fog events that occurred in the first 3-month collection period — nearly 29 kg $ha^{-1}$ as $NO_3$-N and 15–20 kg $ha^{-1}$ as $NH_4$-N (45–49 kg $ha^{-1}$ as total inorganic N). These data suggest that annual N inputs to the forest at CP in years with extensive fog exposure may be higher than previously thought. The data also demonstrate the contrast between throughfall deposition fluxes to the forest floor under mature tree canopies compared to deposition in adjacent open areas. Total inorganic N deposition ($NO_3$-N + $NH_4$-N) under pine canopies with the two types of collectors over the 7 months of exposure was 88–92 kg $ha^{-1}$ at CP and 11–13 kg $ha^{-1}$ at BF. By comparison, bulk deposition in open areas ranged from 5.1–5.4 kg $ha^{-1}$ at CP and 1.9–3.0 kg $ha^{-1}$ at BF (Figs. 3 and 4).

It should be kept in mind that the throughfall values in this study represent deposition rates under the canopies of large ponderosa pine trees only. Average deposition fluxes on the landscape scale will be lower, as canopy cover in this area is approximately 70%[12]. Nonetheless, total deposition inputs at CP are unusually high. The high deposition inputs of N in the western SBM and in parts of the San Gabriel Mountains result in a N saturated condition with the highest streamwater nitrate concentrations from undisturbed watersheds in all of North America[11,14,15,16].

## CONCLUSIONS

Throughfall deposition measurements with the ion exchange resin column collectors were statistically equal to measurements from the conventional throughfall solution collectors. The ion exchange resin collectors hold promise as an inexpensive throughfall monitoring technique that can be applied on a larger scale than otherwise possible with conventional throughfall methods. Throughfall deposition data from the first 7 months of this study suggest that atmospheric N inputs at CP in the western SBM are higher than previously reported[11], especially in years with extensive fog exposure. In some cases, the two methods gave different results for bulk deposition in open canopy-free areas. This appears to be due to a variety of factors including low precipitation volumes, especially during fog episodes, resulting in low sample volumes and inherently high variability in deposition measurements. Additional factors include low replication (n = 4) and animal disturbance of the open samplers, resulting in further reductions in replicates on some dates. High sample volumes and N deposition loadings from fog drip were measured under pine canopies, but as expected, bulk precipitation samplers collect little if any fog in open areas. Further tests are ongoing to test the usefulness of the ion exchange resin column throughfall collector method under varying site conditions in California and with varying times of exposure in the field.

## ACKNOWLEDGEMENTS

The authors gratefully acknowledge the invaluable assistance of Dave Jones, Tony Davila, and Tony Gomez in developing the ion exchange collectors and installing and maintaining the collectors in the field. The efforts of Enrique Jimenez, Joe Decruyenaere, and Diane Alexander with laboratory extraction procedures and chemical analyses are also acknowledged.
